# Denoising MR Images Using Non-Local Means Filter with Combined Patch and Pixel Similarity

**DOI:** 10.1371/journal.pone.0100240

**Published:** 2014-06-16

**Authors:** Xinyuan Zhang, Guirong Hou, Jianhua Ma, Wei Yang, Bingquan Lin, Yikai Xu, Wufan Chen, Yanqiu Feng

**Affiliations:** 1 School of Biomedical Engineering, Southern Medical University, Guangzhou, China; 2 Department of Dermatology, Nanfang Hospital, Southern Medical University, Guangzhou, China; 3 Department of Diagnostic Imaging Center, Nanfang Hospital, Southern Medical University, Guangzhou, China; Institute of Psychology, Chinese Academy of Sciences, China

## Abstract

Denoising is critical for improving visual quality and reliability of associative quantitative analysis when magnetic resonance (MR) images are acquired with low signal-to-noise ratios. The classical non-local means (NLM) filter, which averages pixels weighted by the similarity of their neighborhoods, is adapted and demonstrated to effectively reduce Rician noise without affecting edge details in MR magnitude images. However, the Rician NLM (RNLM) filter usually blurs small high-contrast particle details which might be clinically relevant information. In this paper, we investigated the reason of this particle blurring problem and proposed a novel particle-preserving RNLM filter with combined patch and pixel (RNLM-CPP) similarity. The results of experiments on both synthetic and real MR data demonstrate that the proposed RNLM-CPP filter can preserve small high-contrast particle details better than the original RNLM filter while denoising MR images.

## Introduction

Magnetic resonance (MR) images are usually acquired with low signal-to-noise ratios (SNRs) especially in the implementation of high temporal resolution or high spatial resolution imaging [1], [2]. Meanwhile, some related MR images are often sensitive to noise. A typical example is diffusion-weighted imaging (DWI), which is widely used in routine clinical diagnoses of acute ischemic stroke [3] and various tumors [4], [5], [6], but is often affected by severe noise especially when high *b*-value is applied [7]. Noise in the MR signal is mainly produced from the thermal vibrations of ions and electrons in the receiving coil and the sample [8], resulting in intensity fluctuations of MR images and serious degradation of some clinically useful image information. The following quantitative analysis of MR images through post-processing operations is often degraded by the noise. Thus, reducing noise in MR images is essential and critical to improve image visualization and promote reliability of associative quantitative analysis.

The SNR of MR imaging can be improved by averaging multiple repeatedly acquired images. However, this approach significantly increases data acquisition time and is not feasible in clinical applications where quick procedures are needed. Denoising MR images by various post-processing techniques without heavy computational load has been extensively studied in the past years [9], [10], [11], [12], [13], [14], [15]. A simple approach is the low-pass Gaussian filter [9], which averages spatially adjacent pixels at the expense of blurring. Several approaches that preserve edge information have been proposed and applied to the MR image, including anisotropic diffusion filter [10], [11], wavelet-based filters [12], [13] and total variation [14], with mixed results.

A non-local means (NLM) filter [15], which outputs a weighted average of pixels in a relatively large search window and assigns high weights to pixels with similar neighboring patterns, recently exhibits capability to preserve details and suppress Gaussian-distributed noise as well. Noise in magnitude MR images generally follows a Rician [16] or non-central Chi distribution [17], which has a non-zero mean and causes bias to actual MR images when SNR is low. To address this bias, Manjon et al. proposed an unbiased NLM (UNLM) estimate of MR images in the presence of Rician noise by subtracting the bias from the squared value of the filtered images [18]. A more theoretically reasonable approach is the Rician NLM (RNLM) filter which removes the Rician bias from the average of squared intensities in images [19], [20]. The RNLM approach was also adopted in the later work of Manjon et al. [21], [22]. The NLM algorithm [23] and its Rician-adapted versions [18], [19], [20], [21], [22] show improved denoising accuracy compared with the wavelet and anisotropic diffusion filters when applied to MR images. However, this algorithm and its versions may lead to the blurring or loss of small high-contrast particle details contained in MR images. As these small high-contrast particles may be clinically relevant, the blurring of these particles is generally unacceptable. For example, diffusion-weighted MR imaging is accurate for diagnosing strokes within 6 h of symptom onset [24] and has an important function in diagnosing early cerebral infarctions and monitoring the development of cerebral infarctions [25]. However, the low SNR of diffusion-weighted images might lower the confidence of stroke disease diagnosis. Reducing image noise by post-processing techniques can benefit clinical diagnosis if fine details including the high-contrast particles, which correspond to small infarct lesions, are preserved well in the denoised diffusion-weighted brain images.

As of this writing, to the authors' knowledge, the blurring of high-contrast particles in NLM-based denoising algorithms has not been addressed in previous studies, including MR image denoising. Through extensive experiments, we find that the blurring of sharp particles in NLM-based algorithms is related to the weighting strategy of the central pixel. To avoid over-weighting of the central pixel from very high self-similarity, the weight of the central pixel was proposed to be assigned the maximum weight of the non-central pixels in the search window [15], and this strategy was adopted in the RNLM filter [19], [20], [21], [22]. However, when the intensity of the central pixel is significantly different from those of all other pixels in the search window, that is, when the central pixel cannot find similar pixel in the search window, the aforementioned central pixel weighting strategy [15] causes the reduced contribution of the central pixel to the weighted average output. Thus, small high-contrast particles are unavoidably blurred or filtered out in the denoised MR images.

To retain the small high-contrast particle details in the MR images, we propose a novel weight method using combined patch and pixel (RNLM-CPP) similarity, where only the pixels simultaneously having pixel and neighbourhood similarities will be assigned higher weights in the average. The performance of the RNLM-CPP algorithm is evaluated and compared with the original RNLM filter on both simulated and in vivo MR data.

## Materials and Methods

The MR images acquired for clinical diagnoses were retrospectively selected and anonymized for our denoising research. Our study does not involve new MR scans that are specially performed for the research purpose, thus does not require permit for the local ethics committee. The dataset was acquired for clinical diagnoses and the patient gave his written informed consent for his image to be used for the research purposes and published.

### Denoising Methods

#### NLM Algorithm

An ideal image corrupted with Gaussian noise can be modeled as follows:
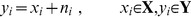
(1)where *i* denotes the pixel index; 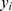 and 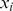 represent the intensity value of pixel *i* in noisy image **Y** and noise-free image **X**, respectively; and 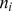 denotes a zero mean Gaussian noise with variance 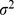.

**Figure 1:**
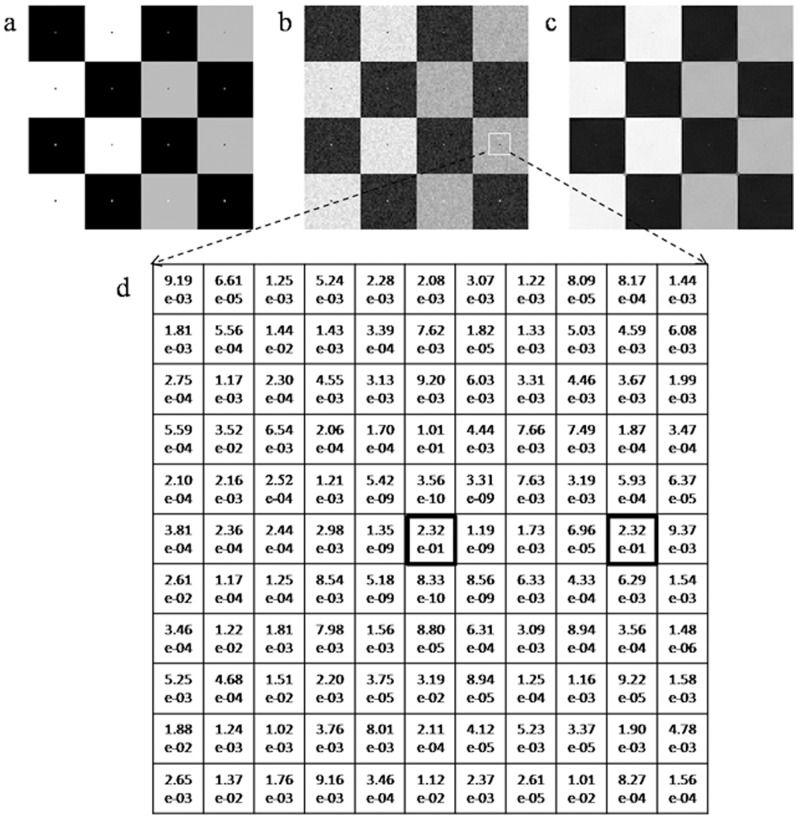
Illustration of NLM filtering effect on small high-contrast particles. (a) Synthesized checkerboard image with one-pixel particle details; (b) Gaussian noise-corrupted image; (c) NLM-filtered image; (d) NLM-calculated weights of pixels in the search window as enclosed by the square in (b).

The NLM algorithm estimates 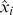 by calculating the weighted average intensity of pixels in a search window 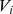 centered at pixel *i* [18], which can be written as follows:
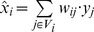
(2)where 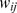 is the normalized weight determined by the distance between the noisy patches located at pixel *i* (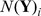) and pixel *j* (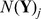) in the noisy image **Y**:



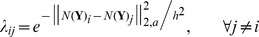
(3)

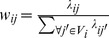
(4)where 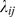 is the weight between pixels *i* and *j* before normalization, 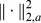 denotes the Gaussian-weighted Euclidean distance, 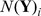 and 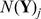 is respectively the vector containing the intensities of the local neighborhood of pixel *i* and *j*, *a* is the standard deviation of the Gaussian function and *h* is the decay rate of weights and controls the degree of smoothing. To avoid over-weighting of pixel *i*, the self-weight 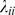 is assigned the maximum weight of non-central pixels in the search window [15], that is,



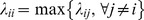
(5)A well-known drawback of the original NLM algorithm is the blurring of sharp small particles in the image. An example is illustrated in **Figure 1**. **Figure 1a** shows a simulated checkerboard image containing one-pixel particle details with different contrast, and **Figure 1b** shows the image corrupted by Gaussian noise. The image filtered by the original NLM algorithm is shown in **Figure 1c**. The contrast between these small particles and their neighboring pixels is reduced, and these particles are difficult to discern in the filtered image. The reason that NLM causes the blurring of small high-contrast particles can be found by inspecting the weights of pixels in the search window around a small particle (**Figure 1d**). The self-weight is set as the maximum weight of non-central pixels (0.232). Given that the sharp particle has an intensity that significantly differs from that of other pixels in the search window, a small self-weight indicates a small contribution from the particle pixel to the final output, which results in blurred or disappeared particles in the filtered image. In theory, assigning self-weight the maximum weight of other pixels in the search window (Eq. (5)) indicates that the central pixel accounts ½ at most for the final output.

#### RNLM Algorithm

The original NLM algorithm was proposed for handling zero mean Gaussian noise. Thus, if applied to denoise MR images, the NLM algorithm should be adapted to deal with non-zero bias caused by Rician noise [16], [26]. An RNLM filter [19] can be written as follows:
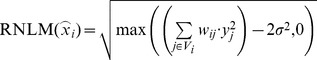
(6)where the parameter 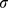 represents the standard deviation of the complex Gaussian noise, and can be estimated from the background region with 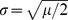, where 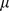 is the mean of squared magnitude in the background of MR images [18].

The current RNLM algorithm uses the maximum weight of non-central pixels as self-weight, thus has the similar particle loss problem as the original NLM algorithm.

To retain the small high-contrast particle details in MR images, we modify the original RNLM algorithm by introducing a novel weight method which uses combined patch and pixel similarity (RNLM-CPP), which is detailed in the following section.

#### RNLM-CPP Algorithm

When small high-contrast particles exist in images, intensity of the particle is generally different from that of the other pixels in the search window to a significant degree. This intensity difference is usually significantly higher than that caused by noise, which can be exploited to overcome the particle-blurring problem of the NLM filter. Thus, a new method for calculating the weight with combined patch and pixel similarity is proposed:
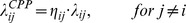
(7)




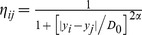
(8)where 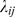 denotes patches similarity between patches located at pixel *i* and *j* as described in Eq.(3), 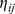 denotes pixel similarity which is defined as a decreasing function of the intensity difference 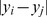 in order to assign high weights to pixels with intensities close to that of the central pixel. In Eq. (8), the parameters 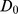 and 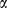 control the position and slope of transition, respectively. The pixel similarity 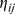 ranges from 0 to 1 and approximates to 0 if the intensity of pixel *j* is significantly different from that of the central pixel *i*. According to Eqs. (7) and (8), only those pixels with high patch and pixel similarity simultaneously are assigned large weights in filtering.

The self-weight can be determined by:
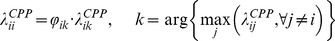
(9)




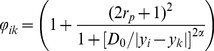
(10)where *k* denotes the index of the non-central pixel that is most similar to the central pixel *i* in the search window, 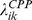 denotes the corresponding maximum CPP weight. In contrast to Eq. (8), the pixel similarity 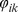 is defined as an increasing function of the intensity difference. In Eq. (10), 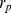 is the radius of the patch window. The weight 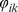 is a scale factor with a range of 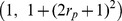, and increases with the absolute pixel intensity difference 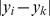. For the small high-contrast particles where the intensity of the central pixel *i* is significantly different from that at pixel *k*, that is, 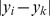 is larger than the factor 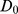, a larger weight (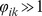) will be assigned to the central pixel. Thus, the particle is selected and preserved.

The adaption of the aforementioned NLM filter with CPP similarity to deal with Rician noise can be formulated as follows:
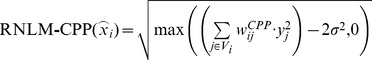
(11)where 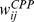 is the weight after normalizing 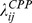.

### Denoising Experiments

#### Experimental Data

To evaluate quantitatively the performance of the RNLM and RNLM-CPP algorithms, simulated MR images from BrainWeb were used [27], [28]. Noise-free T1-weighted (T1w), T2-weighted (T2w), and proton density-weighted (PDw) MR datasets of size 181×217×181 with 1 mm^3^ voxel resolution were downloaded, and Rician noise was added into these datasets based on the formula, 
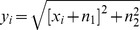
, where 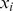 is the noise-free data. 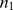 and 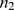 are independent Gaussian distributed random variables with zero mean and standard deviation 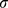. The noise level is defined as the intensity of the brightest tissue divided by 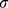. To evaluate the denoising performance under varying noise levels, the MR data with noise levels (1%, 3%, 5%, 7%, and 9%) were synthesized.

To test the performance of the RNLM and RNLM-CPP algorithms on real MR data, two in vivo brain MR datasets were used. The first dataset, downloaded from the fMRI Data Center website (http://www.fmridc.org), was acquired by an MP-RAGE T1w volumetric sequence on a Siemens 1.5 T Vision scanner. The acquisition parameters were repetition time of 9.7 ms, echo time of 4 ms, flip angle of 10°, inversion time of 20 ms, duration time of 200 ms, matrix of 256×256×128, voxel resolution of 1×1×1.25 mm^3^. The second dataset was from a subject with cerebral infarction scanned by an echo-planar/spin-echo DWI sequence on a GE Sigma EXCITE 3.0 T MR imaging system. The dataset was acquired for clinical diagnoses and the patient gave his written informed consent for his image to be used for the research purposes and published. The acquisition parameters were repetition time of 6,200 ms, echo time of 84.7 ms, slice thickness of 5 mm, interslice gap of 1.5 mm, bandwidth of 250 kHz, b-factor of 1,000 s/mm^2^, k-space acquisition matrix of 192×192, image matrix of 256×256, pixel resolution of 0.94×0.94 mm^2^, field of view of 240×240 mm^2^.

#### Implementation of Denoising Algorithms

The 2D versions of the RNLM and RNLM-CPP filters were implemented slice by slice on the synthetic and real 3D MR data. As the performance of the above algorithms depends on the setting of parameters, determining the parameters in the two algorithms is critical. The RNLM and RNLM-CPP algorithms have three common parameters: the radius of search window *r_s_*, the radius of patch window *r_p_*, and the smooth controlling parameter *h*. In all of the experiments, we empirically set *r_s_* to 5, which is a reasonable value for ensuring image quality and filtering efficiency [18]. In the experiments over synthetic data, by tentatively setting the radius of the patch window (*r_p_*) from 1 to 3, we obtained their corresponding optimal *h* values (shown in **Table 1**), which produced maximum peak SNR (PSNR) by an exhaustive search for parameter *h* in a certain range. In the experiments over real brain MR data, the parameters were empirically determined (*r_s_* = 5, *r_p_* = 1 and *h* = 1.2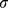) based on the above experiments on synthesized MR data. Aside from the three aforementioned parameters, the RNLM-CPP algorithm has two additional parameters: the cutoff value 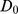, determined as 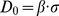 where 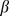 is a constant; and 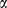, which controls the slope of transition section near 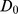. In this study, we tentatively set various 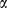 and 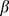 and found their optimal solutions (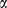 = 4 and 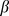 = 5) in the experiments over synthetic data by the PSNR criterion, and transferred these optimal solutions to process real data.

**Table 1:** Optimal *h* values for different denoising algorithms, image types, radius of patches (*r_p_*), and a search window with a radius (*r_s_*) of 5.

	RNLM			RNLM-CPP		
*r_p_*	1	2	3	1	2	3
T1w	1.24σ	1.14σ	1.10σ	1.31σ	1.29σ	1.29σ
T2w	1.23σ	1.19σ	1.16σ	1.32σ	1.36σ	1.38σ
PDw	1.17σ	1.09σ	1.04σ	1.22σ	1.22σ	1.21σ

#### Quantitative Evaluation Measure

PSNR was calculated as a global measure to evaluate quantitatively the performance of the RNLM and RNLM-CPP algorithms on denoising the synthetic MR data. For an image encoded with 8 bits, PSNR can be defined as follows:
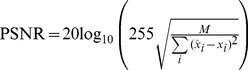
(12)where *M* is the total number of pixels in the denoised image, 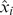 is the estimated intensity at pixel *i* in the denoised image, and 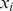 is the true intensity value at pixel *i* in the synthetic noise-free image.

The above PSNR is a global measure which cannot describe well how a filter blurs small details. To provide a quantitative measure of particle preserving, local PSNR (LPSNR) and local structural similarity index [29] (LSSIM) were calculated over local regions around particle details, which were manually located through visual inspection of the noise-free image with size of 5 pixels×5 pixels.

## Results

### Synthetic MR Data

**Figure 2:**
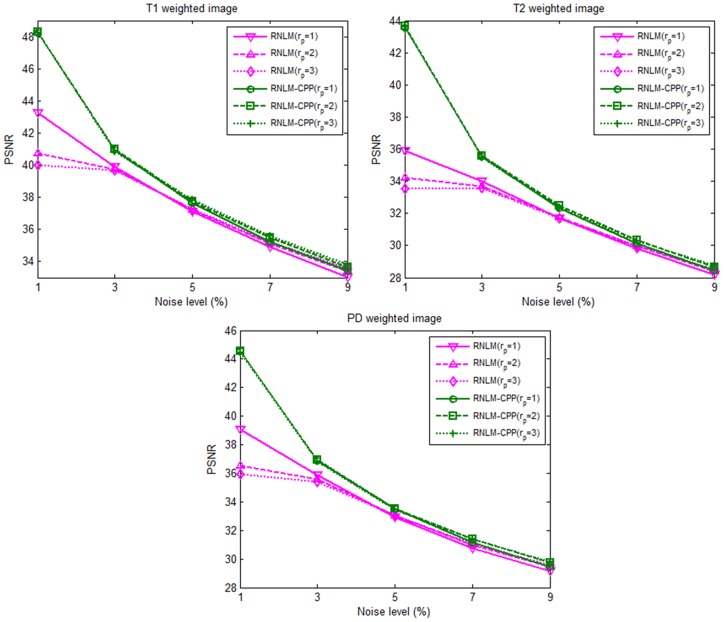
PSNR comparison of RNLM and RNLM-CPP algorithms under varying noise levels (ranging from 1% to 9% with an increase of 2%) for different image types (T1w, T2w, and PD) and patch sizes (radius of 1, 2, and 3).

The optimal *h* values for different patch sizes and image types (T1w, T2w, and PD) with different denoising algorithms are shown in **Table 1**. The PSNR values with optimal *h* against varying noise levels for different *r_p_* and MR image types are plotted in **Figure 2**. A higher PSNR means that the denoised image is much closer to the noise-free image. At low noise level (1%), RNLM-CPP algorithm produced PSNR approximately 4 dB higher than that of the RNLM algorithm. With increasing noise levels, RNLM-CPP consistently produced a slightly higher PSNR than RNLM algorithm.

**Figure 3:**
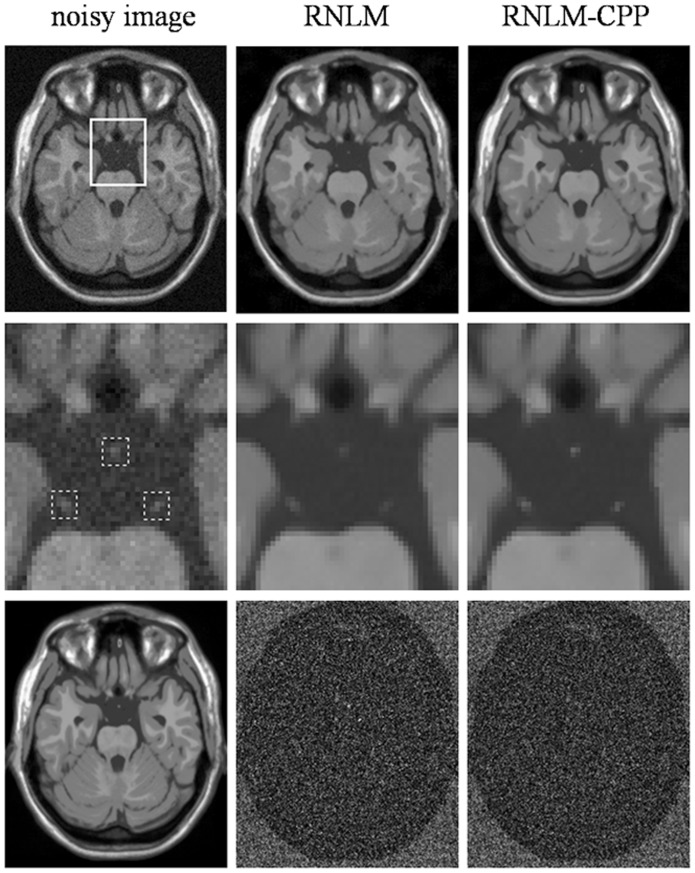
Comparison of RNLM and RNLM-CPP algorithms on denoising simulated T1w images. Top row, from left to right: noisy image with 5% of Rician noise, denoised results with different algorithms. Second row, from left to right: zoomed part of the corresponding images in the top row, the dotted boxes indicate the local areas around manually-defined particles. Bottom row, from left to right: T1w noise-free image and corresponding image residuals.

**Figure 4:**
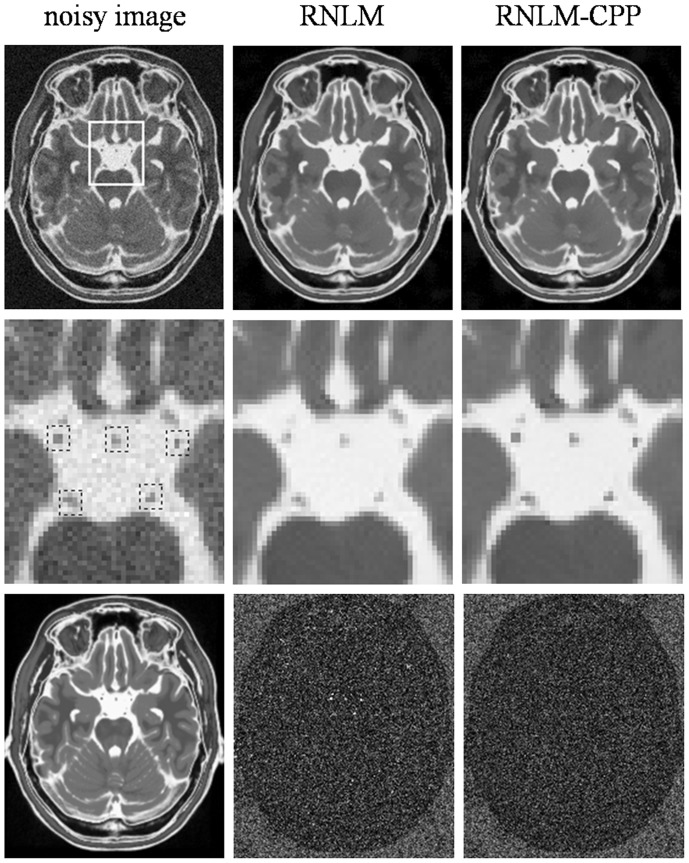
Comparison of RNLM and RNLM-CPP algorithms on denoising simulated T2w images. Top row, from left to right: noisy image with 5% of Rician noise, denoised results with different algorithms. Second row, from left to right: zoomed part of the corresponding images in the top row, the dotted boxes indicate the local areas around manually-defined particles. Bottom row, from left to right: T2w noise-free image and corresponding image residuals.

**Figure 5:**
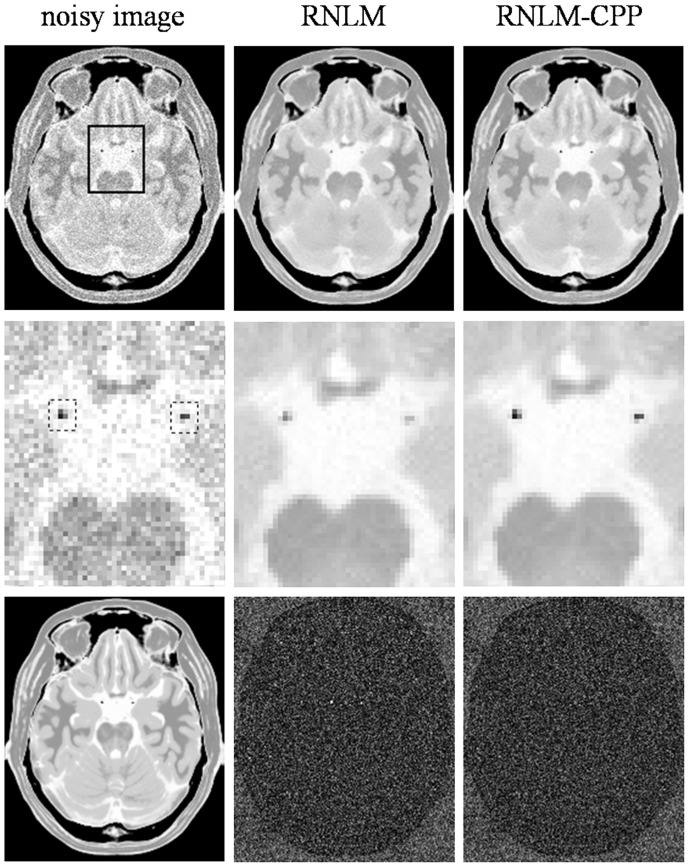
Comparison of RNLM and RNLM-CPP algorithms on denoising simulated PDw images. Top row, from left to right: noisy image with 5% of Rician noise, denoised results with different algorithms. Second row, from left to right: zoomed part of the corresponding images in the top row, the dotted boxes indicate the local areas around manually-defined particles. Bottom row, from left to right: PDw noise-free image and corresponding image residuals.

**Figures 3** **,** **4** **,** **5** show the results of denoising the synthetic brain MR images with particle details (T1w, T2w, and PDw) by RNLM and RNLM-CPP algorithms with the optimal parameters presented in **Table 1** (*r_p_*  = 1, 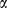 = 4, and 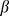 = 5). As shown by the denoised T1w images in **Figure 3**, the RNLM algorithm can suppress noise but causes blurring of small particle details, which can be more clearly observed in the enlarged view. In contrast, the RNLM-CPP algorithm can remove noticeable noise and preserve sharp particle details. Residual images (the absolute difference between denoised and noisy images) further indicate that the RNLM-CPP algorithm has more uniform denoising effect than the RNLM algorithm. This interesting phenomenon can also be observed in the results of denoising T2w and PDw images shown in **Figures 4** and **5**. For a quantitative evaluation of particle preserving, **Tables 2** **and** **3** separately present the LPSNR and LSSIM over the local regions which are delineated by the dotted boxes in **Figures 3** **,** **4** **,** **5** **.** The LPSNR of the RNLM-denoised image was lower than that of the no filtering image, except for the T1w image with noise levels of 7% and 9%. The LSSIM of the RNLM-denoised image was also lower than that of the no filtering image for T1w image with noise level of 1%, T2w image with noise not more than 5%, and PDw image with noise from 1% to 9%. The RNLM-CPP algorithm consistently outperforms the RNLM and no filtering methods in LPSNR and LSSIM.

**Table 2:** LPSNR results for quantitative comparison of RNLM and RNLM-CPP algorithms with parameters (*r_p_*  = 1, *r_s_*  = 5,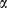 = 4, and 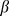 = 5) for T1w, T2w and PDw images.

Data type	Filtering method	Noise level				
		1%	3%	5%	7%	9%
T1w	No filtering	44.83	34.68	30.40	27.20	25.37
	RNLM	33.71	32.73	28.75	28.50	27.58
	RNLM-CPP	**46.12**	**37.81**	**31.87**	**30.92**	**29.23**
T2w	No filtering	39.96	30.58	26.44	23.63	21.05
	RNLM	24.13	24.09	22.24	22.05	20.10
	RNLM-CPP	**40.80**	**31.26**	**28.56**	**26.39**	**22.43**
PDw	No filtering	40.66	31.51	29.68	22.09	21.24
	RNLM	24.02	23.61	23.49	21.19	21.12
	RNLM-CPP	**41.65**	**33.58**	**31.65**	**24.16**	**22.84**

**Table 3:** LSSIM results for quantitative comparison of RNLM and RNLM-CPP algorithms with parameters (*r_p_*  = 1, *r_s_*  = 5, 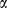 = 4, and 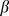 = 5) for T1w, T2w and PDw images.

Data type	Filtering method	Noise level				
		1%	3%	5%	7%	9%
T1w	No filtering	0.9979	0.9806	0.9423	0.9025	0.8747
	RNLM	0.9895	0.9865	0.9705	0.9654	0.9448
	RNLM-CPP	**0.9989**	**0.9940**	**0.9810**	**0.9785**	**0.9634**
T2w	No filtering	0.9993	0.9918	0.9822	0.9686	0.9472
	RNLM	0.9878	0.9853	0.9778	0.9689	0.9661
	RNLM-CPP	**0.9995**	0.9961	**0.9932**	**0.9880**	**0.9825**
PDw	No filtering	0.9992	0.9934	0.9830	0.9575	0.9437
	RNLM	0.9769	0.9737	0.9686	0.9400	0.9365
	RNLM-CPP	**0.9995**	**0.9969**	**0.9956**	**0.9783**	**0.9662**

### Real MR Data

**Figure 6:**
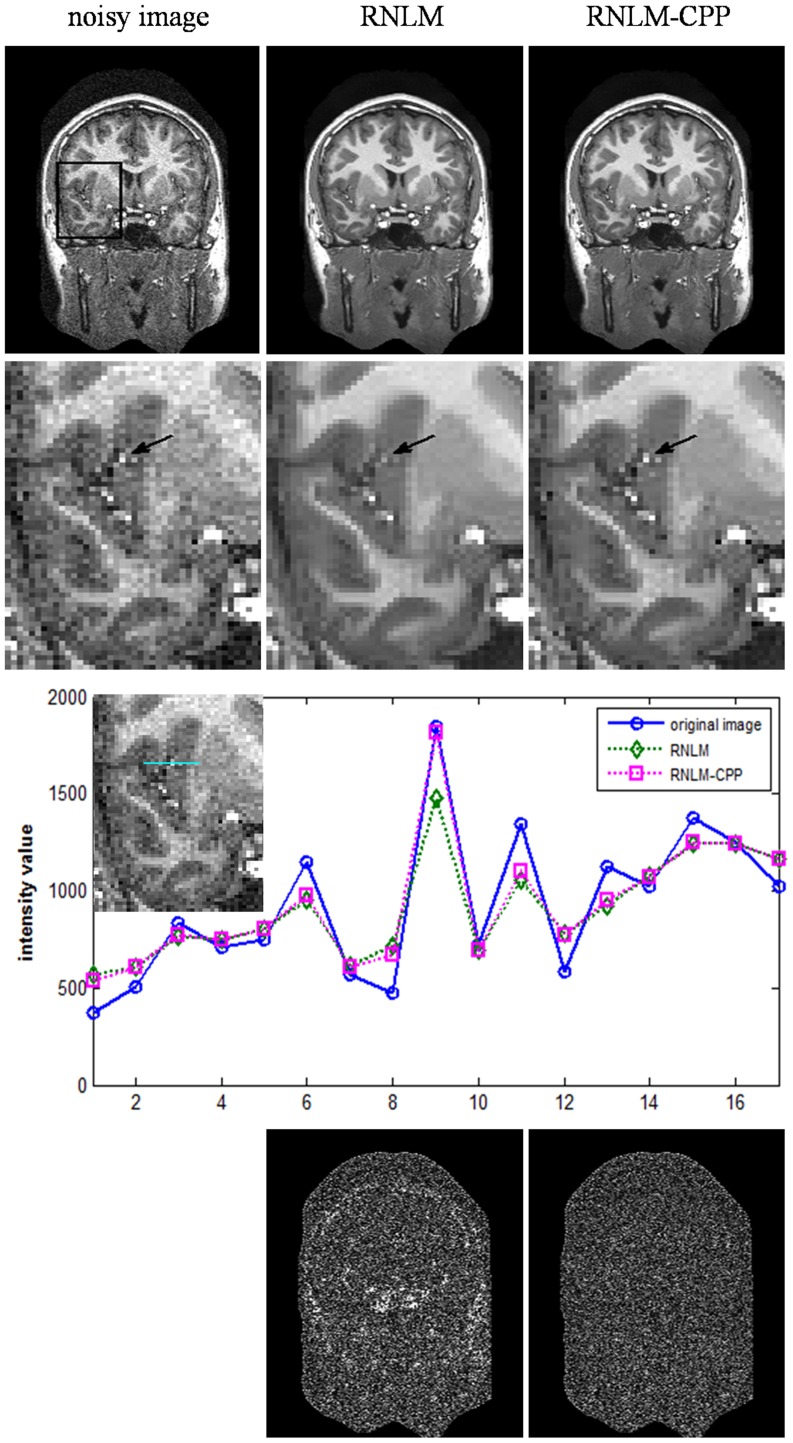
Comparison of RNLM and RNLM-CPP algorithms on denoising real T1 image. Top row, from left to right: real T1w image and denoised results from different algorithms. Second row, from left to right: zoomed image in the square of the top-left image. Third row: intensity profile of noisy image and denoised images located on the cyan line across the particle. Bottom row, from left to right: corresponding residuals.

**Figure 6** shows the results of the denoised T1w brain images by the RNLM and RNLM-CPP algorithms. Both the RNLM and RNLM-CPP algorithms can significantly reduce image noise. The small high-contrast particles of hypointensity signals in the T1w MR images correspond to small vessels in the brain. Although these particles are generally not useful diagnostic information in T1w imaging, they are not noise and should not be blurred or filtered out by the denoising algorithm. However, when focusing on details of the denoised images, as indicated by arrows in the zoomed images (the second row in **Figure 6**), the RNLM algorithm blurred the small particle, but the RNLM-CPP algorithm avoided this drawback. A clearer observation can be made with the intensity profiles of original and denoised images on a line across the particle, as displayed in the third row of **Figure 6**. The residual images in the bottom row of **Figure 6** show that the RNLM-CPP algorithm can reduce noise more uniformly than the RNLM algorithm.

**Figure 7:**
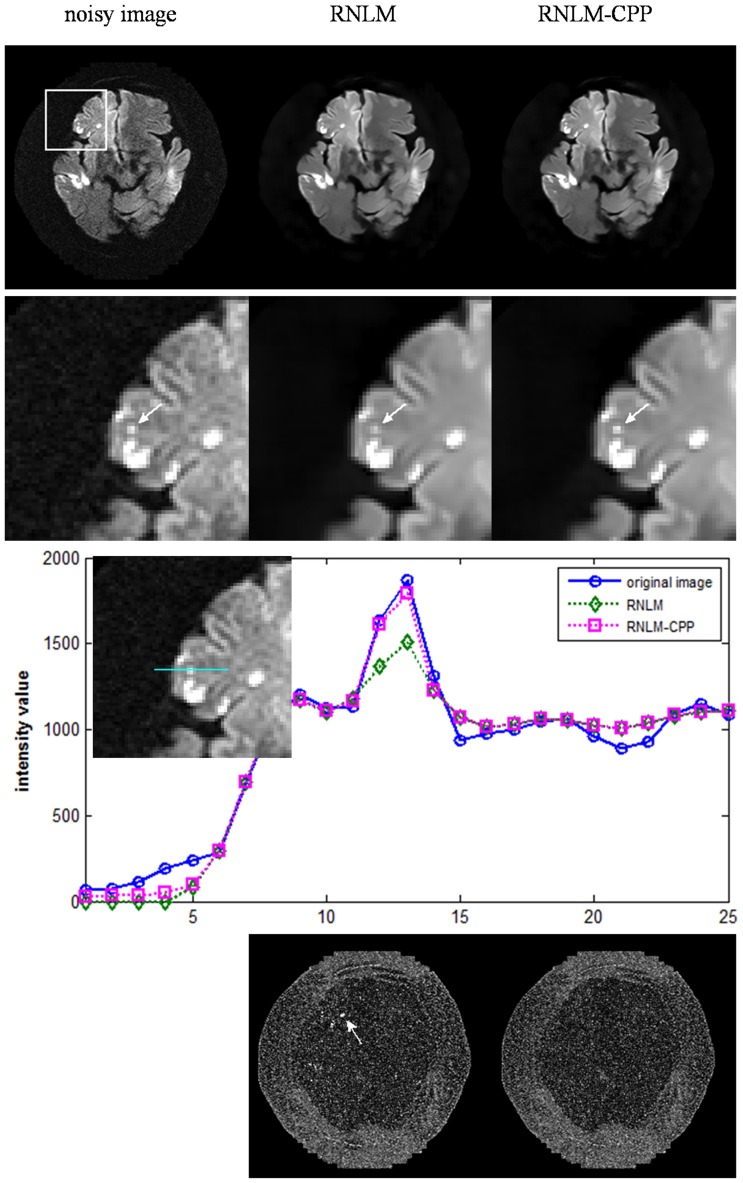
Comparison of RNLM and RNLM-CPP algorithms on denoising real diffusion-weighted image. Top row, from left to right: real diffusion-weighted image and denoised results from different algorithms. Second row, from left to right: zoomed image in the square of the top-left image. Third row: intensity profile of noisy image and denoised images located on the cyan line across the particle. Bottom row, from left to right: corresponding residuals.

**Figure 7** shows the results of the denoised brain DWI data with infarction lesions from a patient with acute cerebral infraction by different algorithms. In diffusion-weighted MR images, the small particles correspond to small infarct lesions, which are useful information for clinically diagnosis, and should be preserved in the denoised images. As shown in **Figure 7**, the original diffusion-weighted image was affected by serious noise, and was significantly denoised by the two algorithms without obvious blurring of details. A high-contrast small particle of infarction lesion in the image, as indicated by arrows in the zoomed images (the second row in **Figure 7**), was blurred by the RNLM algorithm, but preserved well by the RNLM-CPP algorithm. The filtering effect of the two algorithms can be more clearly observed from the intensity profiles along a line across the particle (the third row in **Figure 7**). The residual images of the two algorithms shown in the bottom row of **Figure 7** also illustrate that the RNLM-CPP algorithm shows more uniform denoising effect than the RNLM algorithm.

## Discussion

The NLM algorithm has the ability to reduce noise while preserving details in the image, and its Rician noise version (RNLM) is successfully applied to suppress noise in MR images [18], [21], [23]. However, the traditional RNLM filter generally blurs or even filters out clinically relevant small high-contrast particles, which is a well-known problem that has not been addressed. In this paper, we demonstrated the particle blurring of the RNLM algorithm was caused by setting self-weight as the maximum weight of non-central pixels in the search window. Based on this finding, we proposed a novel RNLM-CPP algorithm using combined patch and pixel similarity. The evaluation results on simulated and in vivo brain MR data showed that the proposed RNLM-CPP algorithm could preserve particles better than the original RNLM algorithm.

The simulation study has the advantage of providing quantitative evaluation of denoising performance. The PSNR, a widely-used measure for evaluating denoisng performance, is dependent on the difference between the whole denoised image and the ground truth, and this difference is mainly from two sources: the blurred particles and the remanent noise after filtering. The remanent noise in the filtered image usually increases with noise levels, and the relative contribution of the blurred particle to PSNR will decrease with noise levels. Thus, at high noise levels (>3%), the PSNRs of the RNLM and RNLM-CPP algorithms become closer. PSNR is a global measure defined on the whole image, thus cannot well reflect the particle preserving performance. For a quantitative evaluation of particle preserving, we resorted to LPSNR and LSSIM defined on local regions of particles. The lower LPSNR and LSSIM of RNLM compared with no filtering in **Tables 2** **and** **3** are because the increased error of particle blurring exceeds the reduced noise error by this filter. Compared with RNLM and no filtering, the higher LPSNR and LSSIM of RNLM-CPP demonstrated that RNLM-CPP can preserve better small high contrast particles while reducing noise.

In practice, high PSNR does not certainly correspond to optimal filtering in the visual perception sense, and quantitative evaluation is generally impossible for real MR images because of the unknown truth. Thus, visual inspection of the filtered images and their residuals is important to evaluate denoising performance. According to visual inspection, the RNLM-CPP algorithm can obtain better particle-keeping performance than the RNLM algorithm in the simulation and in vivo studies (**Figures 3** **,** **4** **,** **5** **,** **6** **,** **7**). In addition, the RNLM-CPP algorithm can yield a result with better spatial uniform effect than the RNLM algorithm in the residual images. The bright area in the residual images of RNLM algorithm mainly corresponds to the blurring of particles.

The proposed RNLM-CPP algorithm identifies high-contrast particles by comparing the intensity of the central pixel with the intensities of other pixels in the search window, based on the assumption that hyperintensity or hypointensity signals are not from noise. This assumption generally holds in magnitude MR images where the noise generally follows a Rician [16] or non-central Chi distribution [17], because the probability that the intensity difference produced by the noise is larger than a threshold (for example, 5 times the SD of noise) is quite small. The pixel similarity was imposed by a predefined function of intensity difference between pixels to distinguish the particles from the noise. The use of other weight functions such as Gaussian, triangular, and hard thresholding, can be investigated in future but beyond the scope of this study. Actually, the adopted function can also be considered as a soft thresholding method, which has an easily adjustable transition region around the threshold. If a hard threholding method is adopted as the function of pixel similarity instead, the proposed method will be equivalent to preselection pixels in the search window based on the intensity difference between the central pixel of two patches, which is designed for particle discrimination and is different from other existent preselection strategies that are based on the mean and the variance of patches and designed to decrease the computational burden [22], [23], [30].

The performance of the RNLM and RNLM-CPP algorithms depends on the setting of filtering parameters: the radius of search window *r_s_*, the radius of patch window *r_p_*, and the smooth kernel *h*. In this study, the optimal parameters were determined by the PSNR criterion from the synthesized brain MR images and transferred to denoising real MR images, in a similar way to that in the UNLM algorithm [18]. Because the true image is unknown, the optimal parameters in denoising real MR data can only be subjectively determined by the visual inspection of denoised images and corresponding residuals. The preliminary results demonstrate that these simulation-determined optimal parameters have the potential to achieve satisfactory performance in denoising real MR data. Further validation of the optimal filtering parameters on a large amount of data by the experienced clinician is required before applying the method in practice.

Preliminary results of the denoised diffusion-weighted images demonstrate that the RNLM-CPP algorithm may benefit the clinical diagnosis of small infarction lesions without the particle-blurring effect in the original RNLM algorithm. The comparisons of performance of the RNLM and RNLM-CPP algorithms under spatially varying noise levels, and their effects on the following image analysis, such as diffusion tensor imaging, fiber tracking, and apparent diffusion coefficient quantification are warranted in future research.

In terms of the computation load, a typical BrainWeb dataset (181×217×181 pixels) took on average 280s for RNLM and 340s for RNLM-CPP, both of which were implemented slice by slice in this study. Thus, the RNLM-CPP algorithm does not substantially increase the computing time compared with the RNLM algorithm. It should be noted that the above time costs were acquired by implementing the denoising algorithms in a 2D fashion, i.e., denoising the 3D data slice by slice. The extension of the proposed algorithm to a 3D version will further improve the denoising performance due to the more robust similarity measure and more similar patterns [18], but will lead to substantially increased computation cost and usually requires several hours to denoise a typical BrainWeb 3D dataset of size 181×217×181. The 3D version of the denoising algorithms can be accelerated by preselecting the similarity pixels and parallel computing [18], [23]. The difference between the 2D- and 3D-particles should be noted since the 2D-particles (particles in the 2D image) may derive from curves in the 3D data besides 3D-particles. There is no obstacle to supposing that the proposed RNLM-CPP algorithm would outperform the RNLM algorithm in preserving 3D-particles.

Since particle blurring is caused by determining self-weight as the maximum weight of non-central pixels in the RNLM algorithm. Other weighting strategy such as calculating the weight of central pixel based on Stein's unbiased risk estimate (SURE) principle [31], [32] may also preserve particles well. However, this SURE-based approach is established in the presence of Gaussian noise, and is not suited for denoising MR images where the noise generally follows the Rician distribution. It should be noted that grouping similar patches into blocks and denoising each block by exploiting sparseness [33], [34] also has the potential to keep well particles and has been demonstrated better denoising performance than the NLM-based algorithms [35]. The comparison of the proposed method with these block-matching and sparseness-based methods was not implemented, since the focus of this study is to address and improve the particle preserving capability of the RNLM algorithm.

In conclusion, the extensive results suggest that the RNLM-CPP algorithm can preserve small high-contrast particle details, which are clinically relevant but usually blurred by the original RNLM algorithm.
